# Application of GRID to Foreign Atom Localization in Single Crystals

**DOI:** 10.6028/jres.105.025

**Published:** 2000-02-01

**Authors:** A. Karmann, W. Wesch, B. Weber, H. G. Börner, M. Jentschel

**Affiliations:** Institut für Festkörperphysik, Friedrich-Schiller-Universitaät, Max-Wien-Platz 1, D-07749 Jena, Germany; Institut Laue-Langevin, BP 156, F-38042 Grenoble, France

**Keywords:** erbium, gamma-spectroscopy, impurity location, lifetime, YAlO_3_

## Abstract

The application of GRID (Gamma Ray Induced Doppler broadening) spectroscopy to the localization of foreign atoms in single crystals is demonstrated on erbium in YAP. By the investigation of the Doppler broadened secondary γ line for two crystalline directions, the Er was determined to be localized on the Y site. Conditions for the nuclear parameters of the impurity atoms used for the application of GRID spectroscopy are discussed.

## 1. Introduction

GRID (Gamma Ray Induced Doppler broadening) spectroscopy is based on the analysis of a Doppler broadened secondary γ line which is emitted from a nucleus highly excited due to neutron capture and recoiled after emission of a primary γ quantum. The Doppler broadened γ line contains information about the lifetime of the excited nuclear states as well as the stopping cross section of the recoiling nucleus in the solid [1.2]. The method has been mainly used in polycrystalline samples to determine the lifetime of nuclear levels and to investigate the interaction of low energy ions with atoms of solids [3,4].

If the recoiling nucleus is embedded in a single crystal its slowing down is non-isotropic. It depends on the surrounding of the recoiling atom and on the crystalline direction. Molecular dynamics (MD) simulations and experiments on single crystals have shown that the energy distribution of the Doppler broadened γ line depends on the orientation of the crystal in relation to the direction of observation [5–8]. Consequently, GRID experiments in combination with MD simulations can give information about lattice positions of foreign atoms as well as about the structural disorder surrounding the recoiling atom.

In the present paper some conditions necessary for the application of GRID spectroscopy are discussed. As an example, a first test of GRID spectroscopy for lattice site localization of Er in yttrium-aluminium-perovskite (YAP, YAlO_3_) is shown.

## 2. Experimental

The applicability of the GRID spectroscopy for materials investigation is restricted to elements having an adequate γ cascade with a sufficiently high intensity after neutron capture, and the lifetime of the intermediate energy level emitting the secondary γ radiation should be in such an order of magnitude that the recoiling nucleus may reach the next, or the next but one neighbouring atom. The Doppler broadening of the secondary γ line of the recoiling nucleus is typically in the order of Δ*E/E* ≈ 10^–6^ to 10^–3^ depending on the primary γ emission and the nuclear mass. And finally, the probability of the γ emission is determined by the thermal neutron flux for the initial capture process and the number of recoiling nuclei per target. The resulting intensity has to be compared with the resolution and the luminosity of the γ spectrometer to get an estimate about the possibility of detecting any structures of the Doppler broadened line profile.

The first GRID experiments on foreign atoms in single crystals were carried out at the Institute Laue-Langevin in Grenoble. The crystals were mounted near the reactor core where a flux of thermal neutrons of 10^14^ cm^–2^ s^–1^ is maintained. The γ line profile was monitored along two crystalline directions with the double flat crystal spectrometer GAMS4 [9] having an energy resolution in the order of Δ*E/E* ≈ 10^–6^ to 10^–4^ depending on the detected *γ* line.

## 3. Results and Discussion

### 3.2 Experimental Results on Erbium in YAP

For the first test of the GRID spectroscopy for foreign atom localization we chose Erbium which is an interesting element for both nuclear and solid state physics. We analyzed the 1649 keV line of ^168^Er (1913 keV → 264 keV) along the [001] and [201] directions. Pretests showed that the 1649 keV transition exhibits a Doppler broadening and that the 1913 keV level has a half-lifetime shorter than 70 fs. The parent ^167^Er has naturally an isotopic abundance percentage of *I*_iso_ = 20 % and a neutron capture cross section of *σ* = 700 b. However, due to the low probability of the chosen γ transition (5×10^–3^ per neutron capture), we had to take a host lattice where a high Erbium concentration can be incorporated. Therefore, we took the laser material yttrium aluminium perovskite (YAlO_3_, YAP with a concentration of natural Erbium in the order of *n* = 10^22^ cm^–3^. The size of the targets was 20×mm×18 mm×2 mm.

**Fig. 1:**
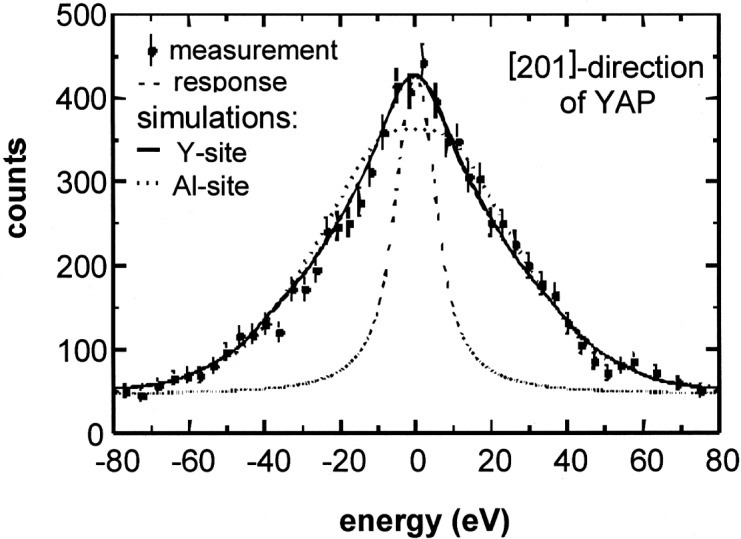
Measured energy distribution of the 1649 keV γ line of ^168^Er in YAP along the [201] direction. the solid and dotted lines represent the calculated line profile for Er on the Y and Al sites, respectively. For comparison with the measured line, the simulated profiles of Fig. 2 and the response function (dashed line of the γ spectrometer are superimposed.

**Fig. 2:**
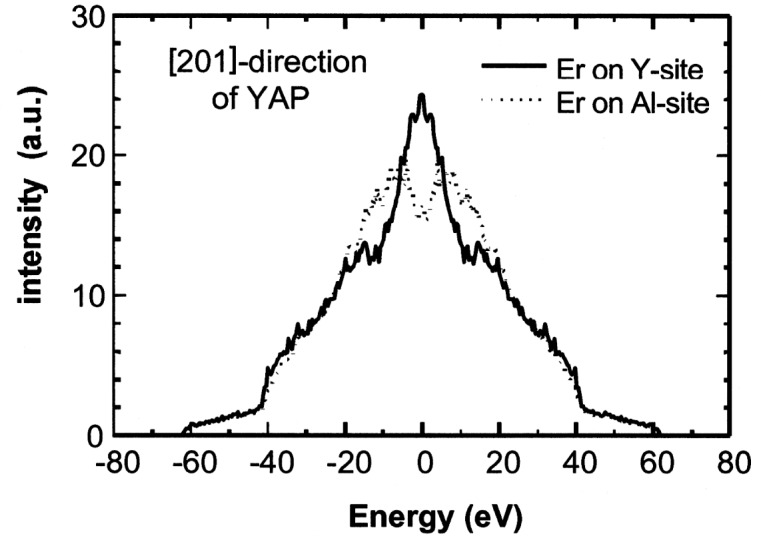
Simulated 1649 keV line of Er in YAP along the [201] direction for Er occupying an Al site or a Y site, respectively.

In Fig. 1, the experimental results for the [201] direction along the Y-Al row (corresponding to the [111] direction in pseudo-cubic description) are compared with the calculated line profiles assuming that Erbium occupies either the yttrium site or the aluminium site. The calculations take into account the energy resolution of the spectrometer by superimposing the response function and the simulated line profiles of Fig. 2. Most of the structures of the line profiles in Fig. 2 are covered by the energy resolution which is low compared to the details of the line profiles. despite of the absence of pronounced structures in the measured line profile, we should show in Ref. [10] the the MD-simulations fit the experiment for both measured orientations if the erbium is assumed to occupy the Y site and the half-lifetime τ_1/2_ of the 1913 keV level is about 20 fs.

From the GRID measurements on Erbium in YAP we deduce the minimum γ intensity required for GAMS4 and the future GAMS5 operating with two curved crystals in DuMond geometry. The minimum intensities listed in Table 1 are estimated under the assumption that the measurement time should not be reasonably longer and the statistical error should be similar. The values obtained for GAMS5 are estimated with the 10^4^ times higher solid angle and the different target sizes [11].

For the application of GRID on impurity atoms, the minimum required impurity density in the crystal is of practical interest. The measured γ intensity is proportional to the γ intensity emitted from the target volume,

$$I_{\text{measured}}\;\propto \sigma \times I_{\gamma }^{2}\times n\times I_{\text{iso}}=\sigma_{\text{eff}}\times n\times I_{\text{iso}}.$$ (1)

*σ* represents the neutron capture cross section *I*_γ_*^2^* the intensity of the secondary γ emission, *n* the density of the incorporated element, and *I*_iso_ the isotopic abundance of the reacting isotope. The product of the neutron capture cross section and the intensity *I*_γ_*^2^* of the secondary γ transition is the effective cross section *σ*_eff_.

The *γ* spectrometer GAMS5 is sensitive to only a thin layer with a thickness of 20 μm. Therefore, it can be applied to layers produced by MBE (Molecular Beam Epitaxy) or to impurity atoms incorporated by ion implantation. for these thin layers, the layer density *D* is a more common quantity, and the measured γ intensity is proportional to

$$I_{\text{measured}}\;\propto \sigma \times I_{\gamma }^{2}\times D\times I_{\text{iso}}.$$ (2)

Taking into account that the maximum density of an element in crystals is about *n* = 5×10^22^ cm^–3^ the effective cross section of the γ emission should be higher than 0.02 b when GAMS4 is used and can be reduced to 5×10^–5^ b for GAMS5 in future. In case of nuclei having transitions with higher effective cross sections *σ*_eff_, its concentration *n* in the crystal could be reduced until the minimum intensity for the used spectrometer is reached.

### 3.2 Selection of Impurity Atoms

Nuclei suitable for the GRID spectroscopy should show γ cascades with a minimum intensity as given above. Additionally, the lifetime of the intermediate level should be in such an order that the recoiling nucleus interacts with the surrounding crystal and influences the structure of the Doppler broadened *γ* line.

For getting a more precise idea about the influence of the lifetime on the Doppler line-profile, we carried out various MD simulations on different crystal lattices and elements. The results of these simulations shows that for the majority of lattice impurity combinations, the influence of the impurity location on the Doppler broadening is most pronounced, if the mean range of the recoiling nucleus is in the order of 2 Å to 3 Å. This mean range corresponds to the atomic bonding length and consequently to the distance to the next neighboring atoms where the recoiling nucleus is scattered.

For mean ranges shorter than 2 Å, the majority of the recoiling nuclei decay before interacting with the next neighboring atoms and the lattice symmetry of the host crystal does not affect the Doppler line.

For mean ranges larger than 3 Å, the stopping of the recoiling nucleus by the next but one neighboring atoms becomes more and more important. The interaction with these atoms causes a further slowing down and reduces the width of the Doppler broadened line. Characteristic structures due to the initial location of the recoiling atom are more and more wiped out [10].

However, for a pre-selection of suitable nuclei, we looked for a simple approximation which takes into account only the nuclear parameters of the recoiling nucleus. We found that the product of the initial velocity of the recoiling atom and the half-lifetime of the intermediate level can be used, subsequently called effective mean range *S*_eff_,

$$S_{\text{eff}}=v_{0}\times \tau_{1/2},\;\text{with}\;v_{0}=E_{\gamma }^{1}/mc.$$ (3)

The initial velocity *v*_0_ is given by the energy *E*_γ_^1^ of the primary γ transition, the mass *m* of the recoiling nucleus, and the light velocity [3].

**Fig. 3:**
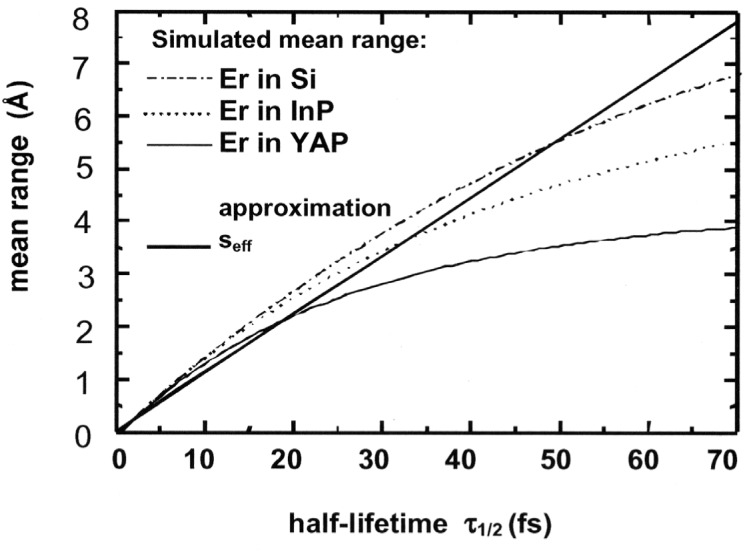
Mean range of the recoiling erbium as a function of the half lifetime of the 1913 keV level. The simulated mean ranges are depending on the crystal where the erbium is incorporated. The straight line indicates the effective mean range *S*_eff_.

Compared to the mean ranges obtained from the MD simulations *s*_eff_ is independent of the used elements of the crystal and it can be calculated easily. For instance, Fig. 3 shows the effective mean range of Erbium as a function of the half-lifetime of the 1913 keV level. Additionally, the mean ranges from simulations of erbium in three different crystals are drawn. The comparison of the ranges shows, that for the most interesting range up to 3 Å, *s*_eff_ is sufficiently close to the simulated mean range to be used as a criterion for the pre-selection.

For *s*_eff_ larger than 3 Å, the difference between the mean range from MD simulation and *s*_eff_ increases depending on the used crystal and the nucleus. A simple estimation is no longer possible.

Taking into account the restrictions concerning the intensity of the γ cascade [Table 1, Eqs. (1), (2)] and the lifetimes of the intermediate level [(Eq. (3)], all known γ transitions listed in the database ENSDF published by the Lawrence Berkeley National Laboratory were analyzed. Additionally, the own results on Erbium ^167^Er(n, γ) from the GRID experiment are included [10].

**Fig. 4:**
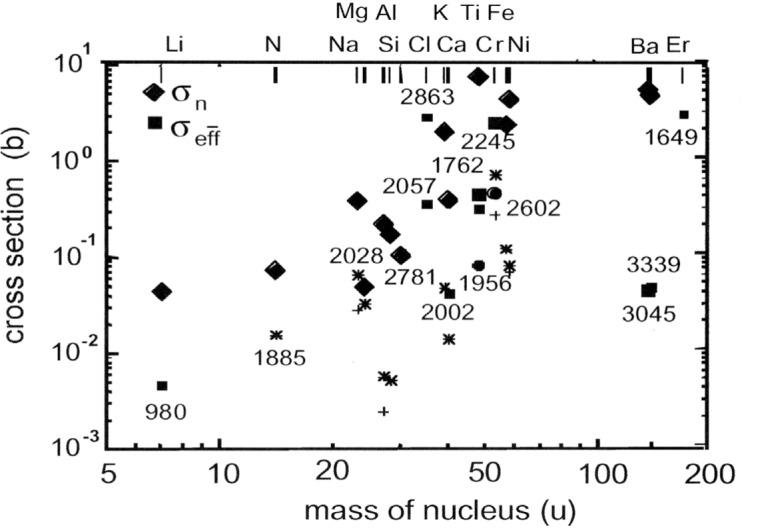
Neutron capture cross sections and effective intensities of the strongest secondary γ transitions belonging to the γ cascades which lead to effective ranges in the range of 1.5 Å to 10 Å. The energy of the most promising secondary γ emissions are indicated in keV.

In Fig. 4 the neutron capture cross sections and the intensities of the secondary γ transitions belonging to the suitable γ cascades of the nuclei are indicated. Some nuclei like Ti show several useful γ cascades. For a better overview, only the energy of the secondary γ transition belong to the most promising γ cascades are indicated in the figure.

**Fig. 5:**
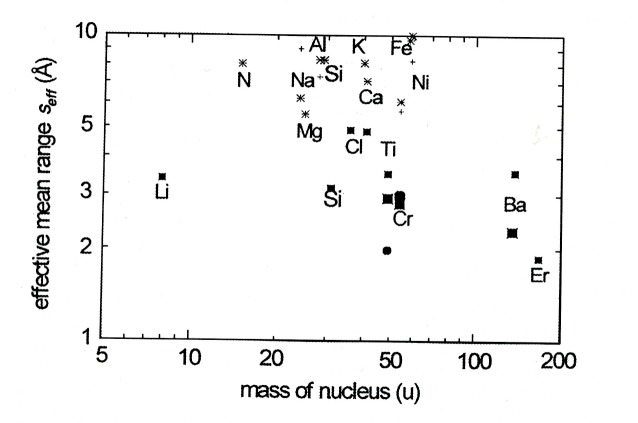
Effective ranges of neuclei before emitting the secondary γ lines shown in Fig. 4.

Figure 5 gives an overview about the effective ranges *S*_eff_ of the nuclei before emitting the γ lines of Fig. 4. Of all known decays, only Si, Ti, Cr, Ba, and Er show γ cascades leading to effective ranges close to the range from 2 Å to 3 Å. All other nuclei like Cl or Fe show longer effective ranges with the consequence to be examined more carefully before application in GRID experiments. The effective range of ^168^Er fits to that required for the GRID experiment and, despite of its low probability, the effective intensity of the 1649 keV transition is one of the strongest which could be found.

Up to now, no suitable γ transition stronger than *σ*_eff_ = 4 b is found in the nuclear data sheets. This restricts the application of GRID to foreign atoms in solids to 2.5×10^20^ cm^–3^ at GAMS4 and to 6×10^17^ xm^–3^ At GAMS5. Especially for heavy nuclei, many γ cascades and lifetimes are not sufficiently known yet. This gives the chance, that the list of suitable nuclei for application of GRID on impurity atoms in crystals will be extended.

## 4. Summary

MD simulations and first experiments on Er in YAP confirm that GRID experiments can be used to localize foreign atoms in crystals. Due to the nuclear parameters and the experimental conditions, the method can be applied only to a very low number of impurity atoms. With the present high resolution γ spectrometers GAMS4 at ILL, the concentration of the impurities should be higher than 2.5×10^20^ cm^–3^. With the GAMS5 spectrometer, which is promised to be available in the near future, a minimum density of 6×10^17^ cm^–3^ will be possible.

## Figures and Tables

**Table 1:** Minimum intensities of the *γ* cascades for the high resolution spectrometer GAMS4 at ILL and the expected limit for GAMS5 in DuMond geometry

Spectrometer	Target size	Minimum *σ*_eff_×*I*_iso_ of the impurity atoms	Minimum *σ*_eff_×*I*_iso_×*n* of the target volume	Minimum *σ*_eff_×*I*_iso_×*D* of the layer
GAMS4	20 mm×18 mm×2 mm^3^	0.02 b	1×10^21^ b/cm^3^	
GAMS5	40 mm×40 mm×0.02 mm^3^	5×10^–5^ b	2.5×10^18^ b/cm^3^	5×10^15^ b/cm^2^
